# Surface plasmon coupling between wide-field SPR microscopy and gold nanoparticles

**DOI:** 10.1038/s41598-023-49583-3

**Published:** 2023-12-16

**Authors:** Qais M. Al-Bataineh, Ahmad D. Telfah, Carlos J. Tavares, Roland Hergenröder

**Affiliations:** 1 Leibniz-Institut für Analytische Wissenschaften-ISAS-e.V., 44139 Dortmund, Germany; 2 Department of Physics, TU Dortmund University, 44227 Dortmund, Germany; 3 Department of Physics, Jordan University of Science and Technology, Irbid, 22110 Jordan; 4 Nanotechnology Center, The University of Jordan, Amman, 11942 Jordan; 5 Department of Physics, University of Nebraska at Omaha, Omaha, NE 68182 USA; 6 Physics Centre of Minho and Porto Universities (CF-UM-UP), University of Minho, 4804-533 Guimaraes, Portugal

**Keywords:** Nanoscience and technology, Optics and photonics

## Abstract

The coupling behavior of the wide field surface plasmon microscopy (WF-SPRM) with single-, two-, and multiple-gold nanoparticles (AuNPs) with different AuNPs sizes is investigated using theoretical, simulation, and experimental approaches. The signal intensity of a single AuNP increases from 208 a.u. to 583 a.u. as particle size increases from 40 to 80 nm, which evidences the signal-building mechanism of Rayleigh scattering theory. A discrete particle model of SPR is used to understand the interaction between an Au-layer and a single AuNP. The calculated intensity profile of the single AuNP from the discrete particle model is accepted with the experimental data. In addition, the superposition between 2-AuNPs surface plasmon waves is studied using the finite element method as well as experimental data from WF-SPRM. The surface plasmon waves around the two particles generate an interference pattern. Finally, it is demonstrated that plasmonic multiple particles scattering can be represented by an effective media, which is described by Maxwell-Garnet equations.

## Introduction

Detecting and characterizing nano-objects with low concentrations, such as biological particles (e.g., viruses and exosome particles), presents a significant challenge in analytical science^1^. Surface plasmon resonance (SPR) stands out as one of the most impressive biodetection techniques, thanks to its various advantages, including label-free detection, high sensitivity, excellent reproducibility, and real-time measurements^2,3^. Moreover, an SPR sensor can be considered as an optical refractometer that examines changes in refractive index near the sensing surface. Consequently, SPR sensors can measure a wide range of binding interactions, such as protein adsorption, ligand-receptor binding, hybridization of RNA and DNA, and nucleic acid detection^4–6^.

Wide-field surface plasmon resonance microscopy (WF-SPRM) follows the principles of SPR based on the classical Kretschmann's scheme^7^. WF-SPRM can detect individual nano-objects in solutions and gases^8–12^. In WF-SPRM, a gold layer is illuminated by a polarized laser beam through a glass prism. The illuminated area is then imaged on a CCD camera to capture high-resolution images of the binding area. Subsequently, a bright spot representing the shape of the detected particle appears in the image, indicating the particle's binding on the surface^13^. The propagation of plasmons by light requires a nano-metal layer on the prism for attenuated total reflection. Several factors influence surface plasmons, including light wavelengths, metal layer thickness and properties, prism properties, and the properties of the surrounding dielectric medium^14^. For ultra-low concentration detection, signal amplification tags are utilized to enhance the sensitivity of SPR detection. Gold nanoparticles (AuNPs) are the most effective tags for enhancing detection due to their unique optical properties and high physical and chemical stability^15^.

A traditional medium model describes the principle of the SPR sensor by treating molecules bound to the sensor surface as an effective medium with an effective refractive index and thickness. However, WF-SPRM can detect individual nano-objects in solutions and gases. Therefore, an SPR discrete particle model is introduced to describe the detection principle for discrete particles. The coupling behavior of WF-SPRM with single, two, and multiple gold nanoparticles (AuNPs) of different sizes (40, 60, and 80 nm) are investigated using theoretical, simulation, and experimental approaches. Furthermore, the finite element method, specifically COMSOL Multiphysics software, is used to examine the interactions between AuNPs of varying sizes and the Au-layer of the SPR sensor. Also, the superposition of surface plasmon waves between two AuNPs using the finite element method is studied, along with experimental data from WF-SPRM. Finally, multiple AuNPs near the gold-sensor surface can be represented through the effective media approximation, specifically the Maxwell-Garnet approximation. The effective dielectric constants are determines for all AuNP sizes to calculate the impact of AuNP size on the sensitivity of the SPR sensor.

## Methods

### Experiment design

The WF-SPRM experiments, based on Kretschmann's configuration^16^, were conducted to detect gold nanoparticles (AuNPs) of different sizes (40, 60, and 80 nm) (Fig. S1)^17–19^. In this experiment, a red (685 nm) laser diode (HL6750MG, Thorlabs GmbH) illuminates the gold-sensor substrate through a glass prism (SF10, n = 1.725) at a fixed incidence angle. The scattered and reflected light is captured through an objective lens (Canon Compact-Macro Lens EF 50 mm $1:2.5$) onto a CMOS video camera (MT9P031, with a resolution of 5 Mp and a pixel size of $2.2\times 2.2$ μm). By varying the incident light angle, the reflected light intensity reaches a minimum, known as the resonance angle.

### Preparation of Au sensor substrate

The gold-sensor substrate is prepared by coating glass slides (SF10, n = 1.725, Helma Optics, Germany) with a Ti adhesive layer (5 nm thickness) and, subsequently, an Au-layer (41–45 nm thickness) using a magnetron-sputtering technique (Innolume). The optical transmittance of the Au-sensor substrate at normal incidence at 685 nm is approximately 4%. Before using the gold-sensor substrate, it undergoes a cleaning process by immersion in a piranha solution (3:1 sulfuric acid (Sigma-Aldrich): hydrogen peroxide (VWR chemicals)) for 5 min, followed by rinsing with distilled water to eliminate traces of the piranha solution, and finally drying with argon gas. For the detection of AuNPs, the Au-sensor substrate is coated with aluminum hydroxide chloride by applying 150 µL of aluminum hydroxide chloride solution (10 wt.%) onto the gold-sensor substrate for 20 min under ambient conditions. Subsequently, the aluminum hydroxide chloride solution is removed, and the Au-sensor substrate is rinsed with distilled water and dried with argon gas.

### Experimental procedures

The Au-sensor substrate is positioned on the base of the glass prism using RI-matching immersion liquid (2 µL, n = 1.725) to match the refractive indices between the glass prism and glass substrate. AuNPs with sizes of 40 nm and a concentration of 7.16 × 10^10^ particles/mL, 60 nm and a concentration of 1.96 × 10^10^ particles/mL, and 80 nm and a concentration of 7.82 × 10^9^ particles/mL were obtained from Sigma-Aldrich. Stock solutions of AuNPs with different sizes were prepared by dilution in water with 0.3% NaCl as follows: 40 nm AuNPs (1:50, 1.431 × 10^8^ particles/mL), 60 nm AuNPs (1:20, 9.80 × 10^8^ particles/mL), and 80 nm AuNPs (1:10, 7.82 × 10^8^ particles/mL). Initially, a sodium chloride buffer solution (NaCl, 0.3%) is pumped through the sensor cell for 1–2 min. After reaching equilibrium, the prism is rotated to the resonance angle (R_*min*_). Starting from the reflectance minimum, the working point is set to 4R_*min*_ within the linear region of the reflectivity curve^20^. Subsequently, AuNPs are introduced into the sensor cell at an average flow rate of 300 μL/min. Within a few seconds, signals arising from the adsorption of AuNPs on the gold-sensor substrate become visible on the video camera, as WF-SPRM visualizes optical patterns generated from the scattering of SP waves on the gold-sensor substrate. Image J software is employed to determine the intensity step signal of the nanoparticle binding event^21^. Additionally, COMSOL Multiphysics is used to design and analyze the proposed model of the SPR sensor using a finite element method-based numerical simulation (Supplementary S2).

## Theoretical analysis

### *p*-polarized light reflectance model

The *p*-polarized light is essential for the sensing of the SPR sensors. The reflectivity (${R}_{p}$) of the SPR sensor can be determined by^22^:

1 $${R}_{p}={\left|{r}_{p}\right|}^{2}$$

2 $${r}_{p}=\left[\left({M}_{11}+{M}_{12}{q}_{5}\right){q}_{1}-\frac{\left({M}_{21}+{M}_{22}{q}_{5}\right)}{\left({M}_{11}+{M}_{12}{q}_{5}\right){q}_{1}}+\left({M}_{21}+{M}_{22}{q}_{5}\right)\right]$$

where ${r}_{p}$ represents the reflection coefficient of $p-{\text{polarized}}$ light and ${M}_{ij}$ are the components of the characteristic matrix, given by^22^,

3 $${M}_{ij}=\prod_{K=2}^{N-1}{M}_{K}=\left(\begin{array}{cc}{M}_{11}& {M}_{12}\\ {M}_{21}& {M}_{22}\end{array}\right)$$

where

4 $${M}_{K}=\left(\begin{array}{cc}{\text{cos}}{\beta }_{k}& -\frac{i{\text{sin}}{\beta }_{k}}{{q}_{k}}\\ -i{q}_{k}{\text{sin}}{\beta }_{k}& {\text{cos}}{\beta }_{k}\end{array}\right)$$

5 $${q}_{k}=\frac{\sqrt{{\varepsilon }_{k}-{n}_{1}^{2}{\text{sin}}{\theta }_{1}^{2}}}{{\varepsilon }_{k}}$$

6 $${\beta }_{k}=\frac{2\pi {d}_{k}}{\lambda }\left(\sqrt{{\varepsilon }_{k}-{n}_{1}^{2}{\text{sin}}{\theta }_{1}^{2}}\right)$$

where $q$ represents the wavenumber, $\beta$ represents optical admittance, ${\varepsilon }_{k}$ and ${d}_{k}$ are the dielectric constant and layer thickness of the ${k}_{th}$-layer, ${\theta }_{k}$ and $\lambda$ are the incident angle and wavelength of the incident light, and $n$ is the refractive index.

### Discrete particle model of SPR

**Figure 1:**
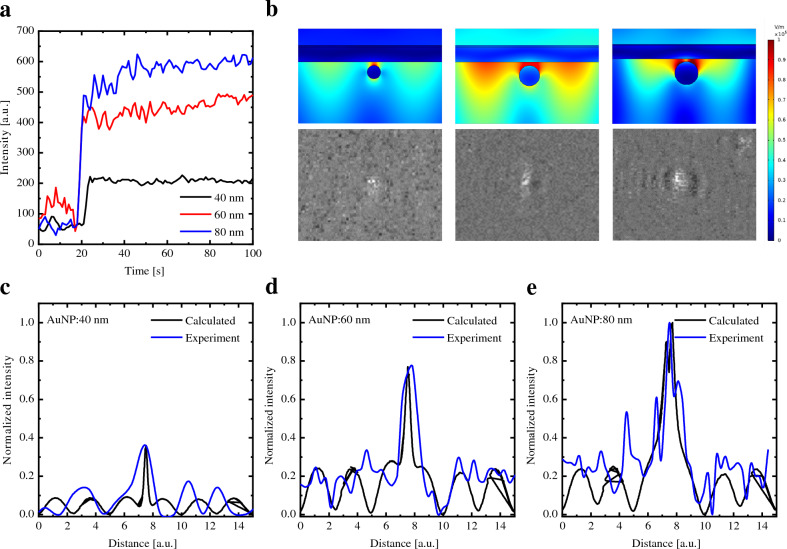
(**a**) Time dependence of the intensity in the middle of the bright spot for AuNPs bound to the Au-layer with different sizes (40, 60, and 80 nm). (**b**) Electric field distribution in the vicinity of AuNPs with different sizes (40, 60, and 80 nm) was simulated using COMSOL Multiphysics and compared to WF-SPRM images. The bright spots are caused by particles bound to the sensor surface. Experimental normalized intensity versus distance compared with the calculated intensity deduced from Eq. (12) for the AuNPs with different sizes: (**c**) 40 nm, (**d**) 60 nm, and (**e**) 80 nm.

Figure 1a shows the detected intensity of the SPR image of the particles arising from the interference between the reflected rays from the Au surface and the scattered plasmons. The incident p-polarized light is partly reflected (${E}_{r}$) at the Au/prism interface and partly transmitted into the Au-layer as an evanescent wave, which excites surface plasmons (${E}_{sp}$)^23^. Initially, the total electric field resulting from the reflected rays and the scattered plasmons primary of the Au/prism systems can be expressed as^24–26^,

7 $$E={E}_{r}{\text{sin}}\left(\omega t\right)+{E}_{sp}{\text{sin}}\left(\omega t+\varphi \right)$$

where $\varphi$ is the phase shift between the background and the radiation generated by the particle. The resulting intensity is given by^18^,

8 $$I=c {\varepsilon }_{0} n {\left|E\right|}^{2}=c {\varepsilon }_{0} n {\left|{E}_{r}{\text{sin}}\left(\omega t\right)+{E}_{sp}{\text{sin}}\left(\omega t+\varphi \right)\right|}^{2}$$

or,

9 $$I=\frac{1}{2}c {\varepsilon }_{0} n\left({E}_{r}^{2}+{E}_{sp}^{2}+2{E}_{r}{E}_{sp}{\text{cos}}\varphi \right)$$

Elastic scattering theory can be employed to study the scattering of surface plasmons by a particle. When the surface plasmon wave is larger than the particle size, the scattered field can be described by a decaying cylindrical plasmonic wave,

10 $${E}_{s}\left(r,{r}{\prime}\right)={E}_{sp}^{0}\left({r}{\prime}\right){e}^{-\kappa \left|r-{r}{\prime}\right|}{e}^{-ik\left|r-{r}{\prime}\right|}$$

where $r$ is the measured location, ${r}{\prime}$ is the particle location. ${E}_{sp}\left({r}{\prime}\right)$ is the surface plasmon field at the location of the particle, $\kappa$ is the decaying constant of the SP, and $k$ is the wave number of SP. Therefore, the total surface plasmon field (${E}_{sp}$) based on the Born approximation is given by^27,28^,

11 $${E}_{sp}\left(r,{r}{\prime}\right)={E}_{sp}^{0}\left(r\right)+\alpha {E}_{sp}^{0}\left({r}{\prime}\right){e}^{-\kappa \left|r-{r}{\prime}\right|}{e}^{-ik\left|r-{r}{\prime}\right|}={E}_{sp}^{0}\left(r\right)+\alpha {E}_{s}\left(r,{r}{\prime}\right)$$

where $\alpha$ is a scattering coefficient related to the polarizability of the particle. ${E}_{sp}^{0}\left(r\right)$ is the SP field in the absence of the particle. The SPR image contrast of the particle is described by,

12 $$I\left(r,{r}{\prime}\right)=\frac{1}{2}c {\varepsilon }_{0} n\left[\left({E}_{r}^{2}+{\left({E}_{sp}^{0}\left(r\right)+\alpha {E}_{s}\left(r,{r}{\prime}\right)\right)}^{2}+2{E}_{r}\left({E}_{sp}^{0}\left(r\right)+\alpha {E}_{s}\left(r,{r}{\prime}\right)\right){\text{cos}}\varphi \right)-\left({E}_{r}^{2}+{E}_{sp}^{2}+2{E}_{r}{E}_{sp}{\text{cos}}\varphi \right)\right]$$

The values ${E}_{r}$ and ${E}_{sp}$ are derived from FEM simulation data. ${E}_{r}$ corresponds to the electric field distribution on the surface of the Au layer in the absence of nanoparticles, while ${E}_{sp}$ corresponds to the electric field distribution on the surface of the Au layer in the presence of nanoparticles. These electric field distributions on the surface of the Au layer represent the experimental data obtained through WF-SPRM measurements on the Au sensor surface.

## Results and discussion

### Single-particle modeling

When AuNPs are bound to an Au surface, the system's symmetry is disrupted. Additionally, plasmon coupling between the AuNPs and the Au surface occurs, resulting in an induced charge in the Au surface that interacts with the AuNPs. However, the polarities of these charges are opposite, leading to an increase in the local electric field between AuNPs and the Au surface due to near-field coupling^29^. An intriguing aspect of WF-SPRM is its ability to detect individual particles bound to the surface. Consequently, the time-dependent relative intensities of AuNPs (40, 60, and 80 nm) binding to the gold-sensor substrate represent three distinct stages: a relatively low, flat intensity before AuNPs binding, a sudden increase in local intensity at the moment of AuNPs binding, and a high, flat intensity after AuNPs binding (Fig. 1a). It is evident that the magnitude of the intensity step is proportional to the size of the AuNPs^30^. The signal intensity increases linearly from 208 a.u. for 40 nm AuNPs to 583 a.u. This clearly demonstrates that the signal generation mechanism fundamentally differs according to Rayleigh's theory^31^. Additionally, the signal noise of AuNPs increases as the size of the AuNPs increases from 40 to 80 nm, which can be attributed to the increasing surface plasmon polaritons produced by the coupling effect between the AuNPs and the Au surface. When the surface plasmon polaritons produced by the Au surface are coupled to the AuNPs, the surface charges between them are redistributed, increasing the local electric field. Figure 1b shows the plasmon coupling occurring between the AuNPs and the Au surface. This coupling results in the generation of a local electromagnetic field when exposed to a 685 nm wavelength laser at an incident angle of 59.8°.

The red regions represent the coupling enhancement regions between AuNPs and the Au surface. 80 nm AuNPs exhibit greater enhancement and confinement than 40 nm AuNPs, indicating that the electric field intensity between the AuNPs and the Au layer increases with increasing AuNPs size. A 40 nm diameter confines incident light between AuNPs and the Au layer in closer proximity to the particle surface than 80 nm AuNPs, indicating a lower efficiency of confined field between AuNPs and the Au layer. This results in efficient excitation of the gap-mode plasmon by 80 nm AuNPs, aligning with the experimental data (Fig. 1b). Furthermore, the presence of AuNPs near the sensor surface disrupts the electric field in the analyte region^32^. Utilizing Eq. 12, we can calculate the intensity distribution of the binding event between the AuNP and the Au surface. Figure 1c–e illustrate the intensity profile distribution for the experiment with AuNPs bound to the Au surface compared to the calculated intensity profile. It is evident that the calculated intensity profile matches the experimental data, indicating the success of our derived model. However, the above simulation assumes a perfect surface, whereas the gold surface has finite roughness. The variation between the calculated and experimental intensities can be attributed to the surface roughness, which is particularly essential for small particle detection compared to the grains^33^.

### Two-particle modeling

**Figure 2:**
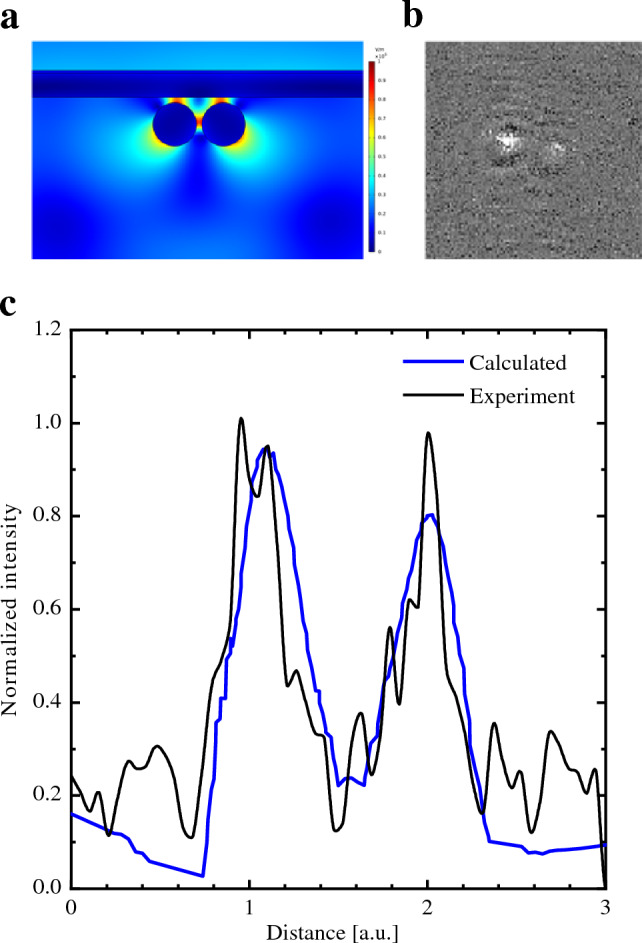
(**a**) Electric field distribution in the vicinity of 2-AuNPs (80 nm). (**b**) WF-SPRM images of 2-AuNPs (80 nm). The bright spots are caused by particles bound to the sensor surface. (**c**) Experimental normalized intensity versus distance compared with the calculated intensity deduced from Eq. (13) for the 2-AuNPs (80 nm).

When two gold nanoparticles (2-AuNPs) are bound to the surface of the Au layer at a distance smaller than the surface plasmon propagation length, the surface plasmon waves around this pair are enhanced due to the constructive and destructive interferences of the surface plasmon waves from each particle (Fig. 2).

Figures 2a,b show the electric field intensity distribution obtained from COMSOL simulations and a WF-SPRM image for two AuNPs with a size of 80 nm. This occurs when they are irradiated with a 685 nm wavelength laser at an incident angle of 59.8°. The surface plasmon waves around the two particles result from the superposition of electric fields between these waves. Combining plasmon intensities of two AuNPs with an initial phase difference (φ) as ${\left|a+a\right|}^{2}$ (or ${\left|2a\right|}^{2}$) produces complex and cumulative superposed plasmon intensities^34^. This can be inferred from Eq. (11), which can be reformulated for two particles:

13 $$I\left(r\right)={I}_{1}+{I}_{2}+c {\varepsilon }_{0} n\left|{E}_{s1}\right|\left|{E}_{s2}\right|{\text{cos}}\left({\varphi }_{1}-{\varphi }_{2}\right)$$

with,

14 $${E}_{sj}={E}_{sp}^{0}\left({r}_{j}\right){e}^{-\kappa \left|r-{r}_{i}{\prime}\right|}{e}^{-ik\left|r-{r}_{i}{\prime}\right|}$$

15 $${I}_{j}\left(r,{r}{\prime}\right)=\frac{1}{2}c {\varepsilon }_{0} n\left[\left({E}_{r}^{2}+{\left({E}_{sp}^{0}\left(r\right)+\alpha {E}_{sj}\right)}^{2}+2{E}_{r}\left({E}_{sp}^{0}\left(r\right)+\alpha {E}_{sj}\right){\text{cos}}\varphi \right)-\left({E}_{r}^{2}+{E}_{sp}^{2}+2{E}_{r}{E}_{sp}{\text{cos}}\varphi \right)\right]$$

Hence, we can compute the intensity distribution of the binding event between the 2-AuNPs and the Au surface. Figure 2c presents the intensity profile distribution for the experiment with 2-AuNPs bound to the Au surface alongside the calculated intensity profile. It is evident that the calculated intensity profile aligns with the experimental data, affirming the success of our derived model. The intensity enhancement observed with the two AuNPs compared to a single AuNP is attributed to the increased local surface plasmon coupling between the AuNPs with the Au surface and each other^35^.

### Multiple-particle model

The multiple particles near the Au layer can be represented by effective media characterized by the effective dielectric constant. The effective dielectric constant of the AuNPs in the analyte, as per the Maxwell-Garnet equations, is provided by^36^:

16 $${\varepsilon }_{eff}\left(\omega \right)={\varepsilon }_{eff}{\prime}+i{\varepsilon }_{eff}^{{\prime}{\prime}}$$

with ${\varepsilon }_{eff}{\prime}$ and ${\varepsilon }_{eff}^{{\prime}{\prime}}$ are the effective real and imaginary parts of effective dielectric constants, respectively, which are given by:

17 $${\varepsilon }_{eff}{\prime}={\varepsilon }_{w}+\frac{f\left({\varepsilon }_{Au}{\prime}-{\varepsilon }_{w}\right)\times \left\{{\varepsilon }_{w}+\beta \left({\varepsilon }_{Au}{\prime}-{\varepsilon }_{w}\right)-f\left(\gamma {\varepsilon }_{Au}{\prime}-{\varepsilon }_{w}\right)\right\}-f{\varepsilon }_{Au}^{{\prime}{\prime}}\times \left(\beta {\varepsilon }_{Au}^{{\prime}{\prime}}-f\gamma {\varepsilon }_{Au}^{{\prime}{\prime}}\right)}{{\left\{{\varepsilon }_{w}+\beta \left({\varepsilon }_{Au}{\prime}-{\varepsilon }_{w}\right)-f\left(\gamma {\varepsilon }_{Au}{\prime}-{\varepsilon }_{w}\right)\right\}}^{2}+{\left(\beta {\varepsilon }_{Au}^{{\prime}{\prime}}-f\gamma {\varepsilon }_{Au}^{{\prime}{\prime}}\right)}^{2}}$$

18 $${\varepsilon }_{eff}^{{{\prime}}{{\prime}}}=\frac{f{\varepsilon }_{Au}^{{{\prime}}{{\prime}}}\times \left\{{\varepsilon }_{w}+\beta \left({\varepsilon }_{Au}^{{{\prime}}}-{\varepsilon }_{w}\right)-f\left(\gamma {\varepsilon }_{Au}^{{{\prime}}}-{\varepsilon }_{w}\right)\right\}-f\left({\varepsilon }_{Au}^{{{\prime}}}-{\varepsilon }_{w}\right)\times \left(\beta {\varepsilon }_{Au}^{{{\prime}}{{\prime}}}-f\gamma {\varepsilon }_{Au}^{{{\prime}}{{\prime}}}\right)}{{\left\{{\varepsilon }_{w}+\beta \left({\varepsilon }_{Au}^{{{\prime}}}-{\varepsilon }_{w}\right)-f\left(\gamma {\varepsilon }_{Au}^{{{\prime}}}-{\varepsilon }_{w}\right)\right\}}^{2}+{\left(\beta {\varepsilon }_{Au}^{{{\prime}}{{\prime}}}-f\gamma {\varepsilon }_{Au}^{{{\prime}}{{\prime}}}\right)}^{2}}$$

where ${\varepsilon }_{Au}{\prime}$ and ${\varepsilon }_{Au}^{{\prime}{\prime}}$ are components of the complex dielectric constant of AuNPs, ${\varepsilon }_{w}$ is the dielectric constant of water, $f$ is the filling factor, defined as the total volume of particles divided by the total volume of the composite analyte, $\beta$ is the shape factor of the particle ($\beta =1/3$ for sphere), and $\gamma$ is a factor, which is given by:

19 $$\gamma =\frac{1}{3{\varepsilon }_{w}}+\frac{K}{4\pi {\varepsilon }_{w}}$$

**Figure 3:**
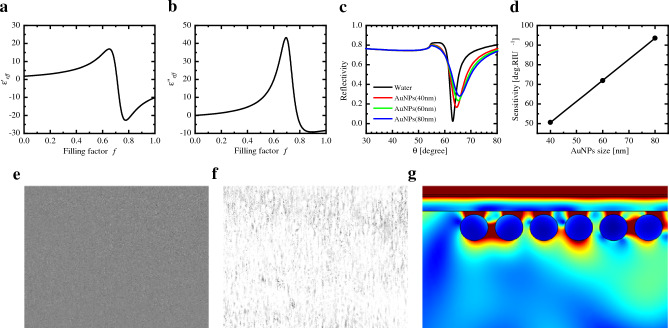
Calculated effective (**a**) real part (${\varepsilon }_{eff}{\prime}$) and (**b**) imaginary part (${\varepsilon }_{eff}^{{\prime}{\prime}}$) of the dielectric constant of AuNPs in the analyte as a function of the filling factor $f \left(0<f<1\right)$. (**c**) SPR reflectivity curve for Kretschmann’s configuration with a constant filling factor ($f=0.02$) for different AuNPs sizes (40, 60, and 80 nm). (**d**) Response of SPR sensitivity as a function of AuNPs size. WF-SPRM images for multiple 80 nm AuNPs (**e**) before binding, (**f**) after binding, and (**g**) Electric field distribution in the vicinity of multiple 80 nm AuNPs.

Here, K represents the interaction parameter between the electric fields generated by adjacent particles. Since the AuNPs in the analyte are present at low concentrations and are sufficiently far apart, the dipolar interaction is negligible, and K equals 0^37^. The calculated values of ${\varepsilon }_{eff}{\prime}$ and ${\varepsilon }_{eff}^{{\prime}{\prime}}$ for AuNPs in the analyte, as a function of the filling factor $f \left(0<f<1\right)$ are depicted in Fig. 3a,b. The ${\varepsilon }_{eff}^{{\prime}{\prime}}$ values increase with an increasing filling factor up to approximately $f=0.7$, and the transition between the positive and negative portions of ${\varepsilon }_{eff}{\prime}$ occurs at $f=0.71$. Consequently, the effective dielectric constant of AuNPs in the analyte exhibits a nonlinear response with respect to the filling factor, consistent with the study by Tamada et al.^36^.

Based on the preceding discussion, we have calculated the theoretical reflectivity curves of the Au layer with different sizes of AuNPs (40, 60, and 80 nm) and a constant filling factor of 0.02 using WINSPALL software (Fig. 3c)^38^. For all AuNP sizes, an increase in the filling factor results in an increase in the incident photon angle at minimum reflectivity (Supplementary S3). At a constant filling factor, the incident photon angle at minimum reflectivity also increases with an increase in the size of the AuNPs (Fig. 3c). The SPR sensitivity is determined as ${\text{S}}={\Delta \theta }/{\Delta n}$, where $\mathrm{\Delta \theta }$ represents the SPR angle shift [°], and Δn is the refractive index change [RIU]. The sensitivity for 40 nm AuNPs is 50.6 deg./RIU, and this sensitivity increases linearly to 93.5 deg./RIU for the detection of 80 nm AuNPs (Fig. 3d). Consequently, larger-sized AuNPs offer improved detection sensitivity. This observation aligns well with the experimental and simulation data presented in Fig. 1b.

Additionally, the WF-SPRM images for multiple 80 nm AuNPs before binding show a distinct dark image (Fig. 3e). After binding multiple 80 nm AuNPs, the image transforms into continuous bright spots, resulting in a brighter image (Fig. 3f). On the other hand, the electric field distribution, derived from COMSOL Multiphysics software, reveals a continuous electric field distribution at the Au surface resulting from the coupling of AuNPs with the Au layer (Fig. 3g).

## Conclusions

The coupling behavior of WF-SPRM with single, double, and multiple gold nanoparticles (AuNPs) of varying sizes is investigated through a combination of theoretical, simulation, and experimental approaches. When AuNPs bind to the Au surface, it disrupts the system's symmetry. Furthermore, plasmon coupling occurs between the AuNPs and the Au surface, leading to the generation of an induced charge in the Au surface, which interacts with AuNPs. The signal intensity of a single AuNP increases from 208 a.u. to 583 a.u. as the particle size increases from 40 to 80 nm, indicating that the signal-building mechanism is based on Rayleigh scattering theory. A discrete particle model of SPR is used to understand the interaction between the Au-layer and a single AuNP. The calculated intensity profile of the single AuNP from the discrete particle model aligns with the experimental data. Additionally, the superposition of surface plasmon waves between two AuNPs is studied using the finite element method and experimental data from WF-SPRM. The surface plasmon waves around the two particles result from the superposition of electric fields between these waves. Combining plasmon intensities of two AuNPs with an initial phase difference ($\varphi$) as ${\left|a+a\right|}^{2}$ (or ${\left|2a\right|}^{2}$) yields a quantum superposition of ${\left|2a\right|}^{2}={\left|a\right|}^{2}+{\left|a\right|}^{2}+2{\left|a\right|}^{2}{\text{cos}}\varphi$, resulting in complex and cumulative superposed plasmon intensities. Finally, the multiple particles near the Au layer are represented as effective media, described by Maxwell–Garnet equations. The sensitivity of 40 nm AuNPs is 50.6 deg./RIU, and this sensitivity increases linearly to 93.5 deg./RIU for the detection of 80 nm AuNPs.

### Supplementary Information

Supplementary Information.

## Data Availability

The datasets used and/or analysed during the current study available from the corresponding author on reasonable request.
