# Supplementary material for: Surface plasmon coupling between wide-field SPR microscopy and gold nanoparticles
Source: Sci Rep. 2023 Dec 16;13:22405. doi: 10.1038/s41598-023-49583-3 (PMC10725443; doi:10.1038/s41598-023-49583-3)
Supplement: Supplementary file 1 — Supplementary Information. [file 41598_2023_49583_MOESM1_ESM.docx]

**Surface Plasmon Coupling Between Wide-field SPR Microscopy and Gold Nanoparticles**

***Qais M. Al-Bataineh^1,2,3*^, Ahmad D. Telfah^4,5,1^, Carlos J. Tavares^6^, Roland Hergenröder^1*^***

*^1^ Leibniz Institut für Analytische Wissenschaften-ISAS-e.V., Bunsen-Kirchhoff-Straße 11, 44139 Dortmund, Germany.*

*^2^ Department of Physics, TU Dortmund University, 44227 Dortmund, Germany.*

*^3^ Department of Physics, Jordan University of Science & Technology, P.O. Box 3030, Irbid 22110, Jordan*

*^4^ Nanotechnology Center, The University of Jordan, 11942, Amman, Jordan.*

*^5^ Department of Physics, University of Nebraska at Omaha, Omaha NE-68182, USA*

*^6^ Centre of Physics of Minho and Porto Universities (CF-UM-PT), University of Minho, 4804-533, Guimaraes, Portugal*

*** ***Correspondence:*** *qais.albataineh@tu-dortmund.de & roland.hergenroeder@isas.de*

**S1. WF-SPRM instrument**


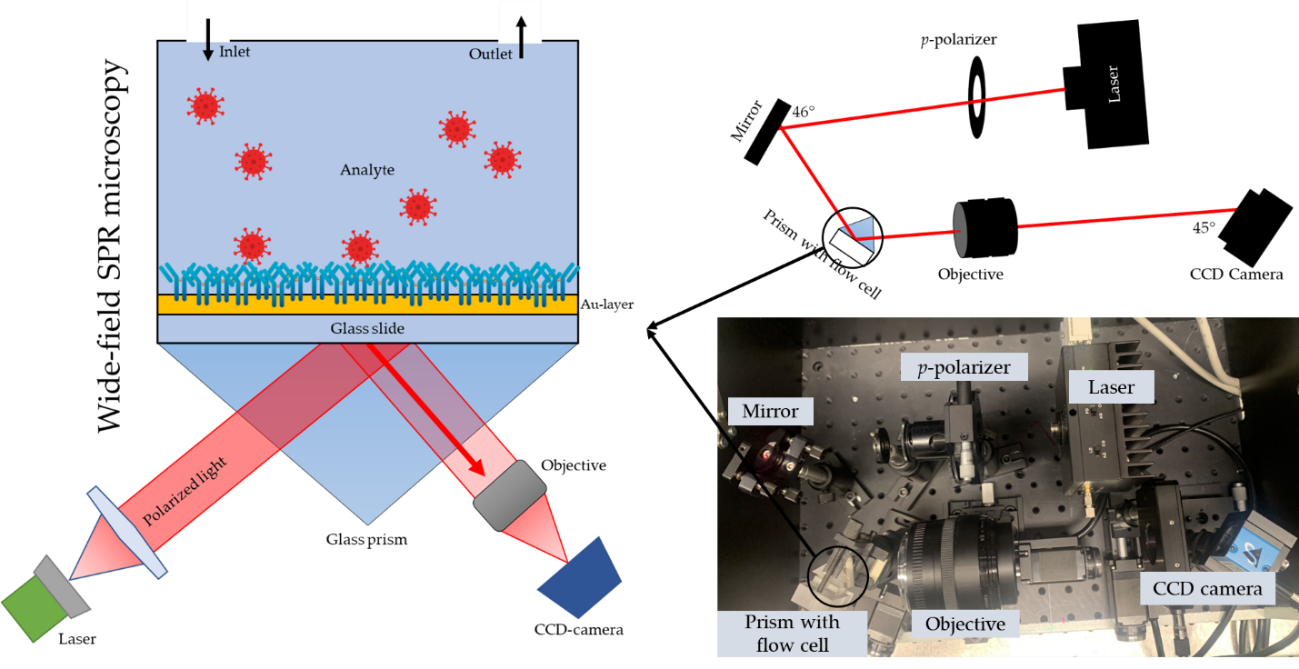


**Figure S1:** Schematic diagram of the experimental home-built design of WF-SPRM based on Kretschmann’s configuration.

**S1. Numerical model**

COMSOL Multiphysics is utilized to design and analyze the proposed model of the SPR sensor (Figure S2a) using finite element method-based numerical simulation. Herein, a 2D geometry based on a prism/Ti-layer (5 nm)/Au-layer (45 nm)/water with Au nanoparticle (40 nm, 60 nm, and 80 nm) located at 10 nm from the Au-layer is proposed (Figure S2b). A light source (λ = 685 nm) was put on top of prism SF11. The AuNPs are placed at 10 nm from the Au-layer, representing the thickness of binding molecules. In addition, Floquent periodicity and periodic port conditions are also applied (Figure S2b, red lines). Before starting the simulation, the simulated SPR sensor is divided into small areas (meshes). In this work, the mesh is chosen to be a free triangular element with a size ranging between 6.09 x 10^-5^ µm and 0.03 μm (Figure S2c). In addition, the angular interrogation is performed by variating the incident angle of a light source from 20° to 89° with 0.1° incremental deviation using a parametric sweep operation. The reflectivity is calculated for all angles, and the resonance angle is detected at minimum reflectivity.


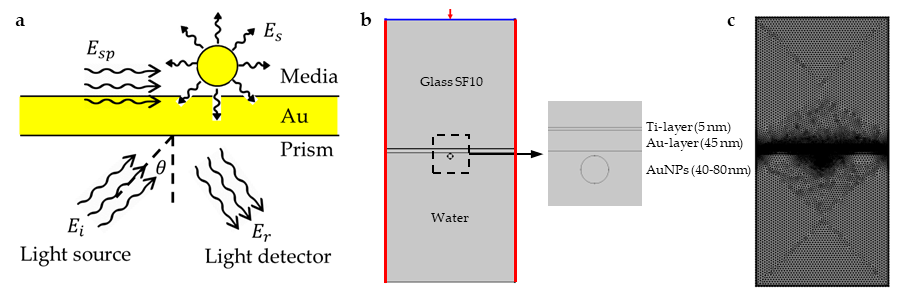


**Figure S2: (a)** 2D illustration of the experimental design of WF-SPRM based on Kretschmann’s configuration. **(b)** COMSOL Multiphysics view structure and **(c)** computational meshing domain for proposed 2D geometry based on prism/Ti-layer (5 nm)/Au-layer (45 nm)/water with Au nanoparticle (40 nm, 60 nm, and 80 nm) located at 10 nm from the Au-layer.

**S2. Multiple-Particle Interference**

**Figure S3:** SPR reflectivity curve for Kretschmann’s configuration with AuNPs size of 40 nm and different constant filling factor ($f=0.02-0.16$).

**Figure S4:** SPR reflectivity curve for Kretschmann’s configuration with AuNPs size of 60 nm and different constant filling factor ($f=0.02-0.16$).

**Figure S5:** SPR reflectivity curve for Kretschmann’s configuration with AuNPs size of 80 nm and different constant filling factor ($f=0.02-0.16$).
